# How Do Microbial Pathogens Make *CEN*s?

**DOI:** 10.1371/journal.ppat.1002463

**Published:** 2012-02-09

**Authors:** Kaustuv Sanyal

**Affiliations:** Molecular Mycology Laboratory, Molecular Biology & Genetics Unit, Jawaharlal Nehru Centre for Advanced Scientific Research, Jakkur, Bangalore, India; Duke University Medical Center, United States of America

## What Is the Link between Drug Resistance and Aneuploidy in Microbial Pathogens?

In a variety of pathogens, drug resistance and aneuploidy are intimately associated. Anueploidy is believed to alter the dosage of certain genes that can impart drug resistance. Generation of a new chromosome by duplication of chromosome segments followed by telomere addition in a pathogenic yeast *Candida glabrata* [1] and isochromosome formation by breakage of chromosome 5 at the centromere followed by joining two identical arms of chromosome 5 in an opportunistic yeast *Candida albicans* [2] have been shown to occur frequently in drug-resistant isolates. Fluconazole-resistant strains of another pathogenic fungus, *Cryptococcus neoformans*, have been shown to be disomic for certain chromosomes [3]. Experimental evidence suggests that acquisition of chromosomes by the plant pathogenic fungus *Fusarium* can convert a non-pathogenic strain to a pathogenic one [4]. Anueploidy has been shown to be the cause of drug resistance in the protozoan parasite *Leishmania* as well [5].

Improper chromosome segregation is one route to aneuploidy. The centromere–kinetochore complex facilitates interaction between a chromosome and the spindle microtubules to ensure equal segregation of chromosomes from a mother to daughter cells. In addition to serving as sites of protein assembly to form kinetochores, centromeres also hold two sister chromatids together until the onset of anaphase by balancing opposing forces acting on them—a pole-ward (outward) force generated by spindle microtubules depolymerizing toward opposite poles, and a cohesive (inward) force between two sister chromatids. Paradoxically, in spite of performing the conserved function of chromosome segregation, centromere (*CEN*) DNA sequence and organization of *CEN* DNA elements vary widely among eukaryotes. In contrast, several kinetochore proteins are evolutionarily conserved, although sometimes this conservation is restricted to a group of organisms. For example, the Dam1 complex, an outer kinetochore protein complex, is fungus specific and is essential for viability in *C. albicans* [6], [7]. Thus, this complex may be a suitable target for development of anti-fungal drugs. However, a *CEN*-specific histone H3 variant of the CENP-A/Cse4 family is found to be universally associated with the formation of specialized and unique chromatin at all functional *CEN*s despite seemingly diverse *CEN* DNA sequences.

## What Are the Different Types of *CEN*s in Eukaryotic Pathogens?

Some organisms have holocentric chromosomes where the *CEN* is diffused and *CEN* elements are distributed throughout a chromosome. However, most organisms have monocentric chromosomes where a *CEN* is localized to a single region on a chromosome. Monocentric *CEN*s are of three types: a) “point” *CEN*s in which centromere function is retained in a short (<400 bp) stretch of DNA with highly conserved protein binding motifs and often a single CENP-A-containing nucleosome, b) “small regional” *CEN*s with CENP-A-containing chromatin formed on a stretch of DNA (<40 kb) that is longer than the point centromere but often lacks any protein binding sequence motifs or conserved pericentric repeats, and c) “large regional” *CEN*s that span a long region (>40 kb–a few Mbs) usually rich in repeats and often CENP-A-containing nucleosomes are interspersed with H3-containing nucleosomes. Formation of a kinetochore on most point *CEN*s initiates with binding of a DNA sequence-specific kinetochore protein [8]. In contrast, several lines of evidence suggest that epigenetic factors, not the DNA sequence alone, also contribute to centromere identity in organisms carrying small or large regional centromeres [8]–[10].

## How Are Centromeres Organized in Fungal Pathogens?

**Figure 1:**
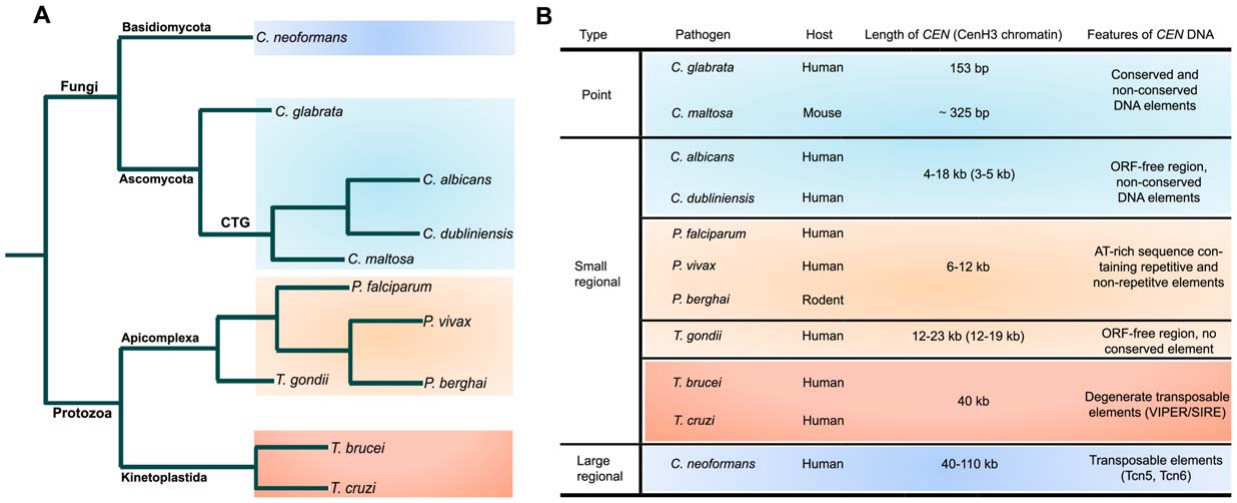
Structural organization of centromeres in various microbial pathogens. (A) A schematic showing relatedness of various microbial pathogens. (B) A table summarizing essential features of different types of centromeres identified in microbial pathogens.

The pathogenic fungi show wide diversity in *CEN* structure (Figure 1; reviewed in [11]). *C. glabrata*, a human pathogenic ascomycetous yeast, has short point *CEN*s. *CEN*s in *C. glabrata*, like the well-studied *CEN*s of baker's yeast *Saccharomyces cerevisiae*, have three *CEN* DNA elements (CDEs). CDEI (8 bp) and CDEIII (18 bp) are well conserved in all the chromosomes, while CDEII (77–79 bp) is not conserved in sequence but is highly AT-rich (83%–93%) [12]. A circular mini-chromosome containing a short *CEN* sequence (<160 bp total, inclusive of CDEs) and a replication origin/autonomously replicating sequence (*ARS*) can stably propagate through many generations. Both CDEI and CDEIII are required for proper function of a centromere. CDEIII has the crucial CCG sequence and certain mutations in CDEIII result in a complete loss of centromere function. *Candida maltosa*, a hemiascomycetous yeast that is phylogenetically closely related to other human pathogenic *Candida* species of the CTG clade such as *C. albicans*, is believed to be non-pathogenic to humans but has been reported to be virulent occasionally when tested in the mouse model [13]. Although a 325-bp *C. maltosa CEN* region with a conserved CDEI and an AT-rich CDEII sequence can provide mitotic stability to an otherwise unstable *ARS* plasmid, the conserved CDEIII sequence, frequently found in point centromeres, is absent [14], [15].

*C. albicans*, the most common fungal pathogen isolated from patients, and *Candida dubliniensis*, a less frequently observed but closely related species, have small regional *CEN*s. *C. albicans CEN*s were identified as binding sites of Cse4, a CENP-A homolog [16]. Each of the eight *CEN*s has a 3–5-kb CENP-A-rich sequence present in a 4–18-kb ORF-free region [17]. Only chromosome 5 has long inverted pericentric repeats. The AT-richness at the *CEN* region is similar to the overall AT-content of the genome. *CEN* formation in this organism requires a pre-existing epigenetic memory, and thus an exogeneously introduced *CEN/ARS* plasmid is mitotically unstable in *C. albicans* [18]. Strikingly, when a native centromere is replaced by a transcribed, selectable gene, the acentric chromosome often is stabilized by forming a neocentromere very efficiently elsewhere on the chromosome on a region with no sequence homology to the native centromere [19]. *CEN*s in *C. dublineinsis* have been identified by synteny analysis with *C. albicans* and are found to be rich in CENP-A binding [20]. Although all the features of Cd*CEN*s are very similar to those of Ca*CEN*s, *CEN* DNA sequences in these two organisms were found to be rapidly evolving. The *CEN* sequence of another pathogenic yeast, *Candida lusitaniae*, has been identified by bioinformatic analysis but not experimentally verified [21]. A GC-poor trough approximately 4 kb in length found in every chromosome is the putative centromere in this organism.

A basidiomycetous fungus, *C. neoformans*, which causes fungal meningitis in humans, has been predicted to have long regional centromeres. Transposon (Tcn5, Tcn6)-rich sequences, located within 40–110 ORF-free regions that are present once per chromosome, are the presumptive *CEN*s [22]. Moreover, measurement of meiotic recombination rates by random spore analysis also indicates that *URA5* and *ADE2* are *CEN*-linked in agreement with the physical map [23]. These *CEN*s are similar to those of the opportunistic pathogen *Aspergillus nidulins*, an ascomycete [24].

## How Are Centromeres Organized in Ancient Eukaryotic Pathogens, the Protozoan Parasites?

Topoisomerase II (Topo-II) has been implicated in chromosome segregation in a range of organisms from yeast to humans [25], [26], [27]. After DNA replication in S phase, sister chromatids remain attached together partly by strand catenation at centromeres as cells enter mitosis. During sister chromatid separation, Topo-II decatenates by creating double-strand DNA breaks followed by passage of uncut DNA through breaks and ligation to repair the breaks. Topo-II has even been implicated to be an epigenetic marker for kinetochore assembly [28]. Etoposide, a Topo-II inhibitor, blocks the ligation step and results in DNA breaks at Topo-II binding sites [29]. In the malaria parasite *Plasmodium falciparum*, an apicomplexan, an etoposide-mediated Topo-II cleavage site was mapped to a single 10-kb region in each of the 14 chromosomes [30]. Further analysis shows that, with the exception of chromosome 10, this site corresponds to a 6–12-kb ORF-free region with a highly AT-rich region of 2.3–2.5 kb that contains a repetitive region and a core region. Both the core and the repeat region are important for centromere function. Various repetitive DNA elements present in these regions do not show any interchromosomal conservation, but nucleotide content (AT-richness) and the size of these putative *CEN*s are strictly conserved. Another human malaria parasite, *Plasmodium vivax*, has AT-rich syntenic regions in three chromosomes [30]. Thus, both these *Plasmodium* species have centromere properties similar to the small regional centromeres found in *C. albicans* and *C. dubliniensis* (Figure 1). Based on the syntenic locations of Pf*CEN*s, centromeres were identified in genetically tractable *Plasmodium berghei*, which causes malaria in rodents [31]. A 1.2-kb highly AT-rich (96%) sequence found in Pb*CEN5* also contains a non-repetitive core and a repetitive region. Centromere function was formally demonstrated by cloning this region in a plasmid. The Pb*CEN5* plasmid was evenly segregated and was stably maintained at low copy number in 90% of the parasites 21 days post-transfection in the absence of drug selection for the plasmid. A linear *Plamodium* artificial chromosome (L-PAC), constructed by adding telomeres to the *CEN* plasmid, was also stably maintained like a natural chromosome. L-PAC exhibited a 10- to 100-fold increase in transfection efficiency as compared to the circular *CEN* plasmid. In another apicomplexan, *Toxoplasma gondii*, centromeres were identified as binding sites of the centromeric histone CENP-A [32]. One CENP-A binding region per chromosome was identified in 12 of the 14 chromosomes. CENP-A binding regions were restricted to 16±3.4 kb sequences that are present in 17±5.6 kb regions largely devoid of ORFs. Etoposide-mediated cleavage in these regions further confirmed centromere identity. Again, no obvious sequence bias or conserved sequence elements were detected, suggesting *Toxoplasma* centromeres belong to the class of short regional centromere (Figure 1). Centromeres have been identified in two kinetoplastid protozoans, *Trypanosma cruzi* and *Trypansoma brucii*, the causative agents of Chagas disease in Latin America and of sleeping sickness in humans and nagana in cattle in sub-Saharan Africa, respectively (Figure 1). By telomere-mediated chromosome truncation, a 16-kb GC-rich transcriptional “strand switch” domain was identified as the centromere in *T. cruzi* [33]. Etoposide cleavage analysis also confirmed that a Topo-II binding site colocalizes to this single locus on *T. cruzi* chromosome I. This 16-kb region has degenerate retroelements (VIPER/SIRE) and non-LTR transposons but does not contain any satellite repeats. This region is also flanked by transcriptionally quiescent polycistronic satellite units. *T. brucii* also has a similar 11-kb GC-rich domain in a syntenic region between directionally oriented gene clusters that contain degenerate retroelements (DIRE) and a 5.5-kb AT-rich stretch with 58-bp degenerate repeats [34].

## What Are the Factors That Determine *CEN* Identity?

Since centromeres in certain pathogenic budding yeasts form in a DNA sequence-dependent manner while in most other microbial pathogens centromeres form in a sequence-independent manner, a universal mechanism determining centromere identity seems to be improbable. The best example of DNA sequence-independent assembly is the formation of neocentromeres on non-native loci with no apparent sequence similarity when a native *CEN* is deleted or inactivated. Centromeres are found to be clustered and occupy a distinct locus at the nuclear periphery in most yeasts, including *C. albicans* [17] and *C. dublineinsis* [20], as well as in the protozoan parasite *T. gondii* [32]. A three-dimensional higher order chromatin structure (clustered centromeres) that occupies a favorable nuclear space (such as the one with high CENP-A concentration) may be the determining factor for *CEN* identity in these organisms. *CEN*s have been shown to replicate earliest in S phase in *C. albicans* [35], which indicates that replication timing can be a determinant of *CEN* identity. Certain post-translational modifications of canonical histone H3 can also support *CEN* formation [36]. Di- and tri-methylated Lys 9 of histone H3 (H3K9Me2/H3K9Me3), marks associated with pericentric heterochromatin, have been shown to be enriched at centromeric regions of *T. gondii* [32]. Interestingly, although H3K9Me3 molecules are enriched in transcriptionally silent loci, centromere regions in *P. falciparum* are largely devoid of such molecules. Non-coding RNA (ncRNA) and the RNAi machinery also play a role in centromere formation in certain eukaryotes [36]. In *P. falciparum*, short ncRNAs (75–175 nucleotides) transcribed from both strands of *CEN* regions localize to the nucleus [37] even though *Plasmodium* species lack components of the RNAi machinery. *T. bruceii* is the first protozoan parasite in which RNAi has been shown to be functional [38]. Disruption of RNAi causes defects in chromosome segregation in this organism [39]. Strikingly, the related species *T. cruzi* does not have RNAi machinery. Thus, a variety of factors, often species specific, may contribute to formation of a functional *CEN*, but elucidation of a specific mechanism that determines *CEN* identity in most pathogenic eukaryotes is a puzzle to be solved. Understanding centromere-kinetochore structure-function of these pathogens can help us develop specific drugs with fewer side effects to combat microbial infection.
